# Measuring and understanding the effects of a performance based financing scheme applied to nutrition services in Burundi—a mixed method impact evaluation design

**DOI:** 10.1186/s12939-016-0382-0

**Published:** 2016-06-14

**Authors:** Manassé Nimpagaritse, Catherine Korachais, Dominique Roberfroid, Patrick Kolsteren, Moulay Driss Zine Eddine El Idrissi, Bruno Meessen

**Affiliations:** Health Economics Unit, Department of Public Health, Institute of Tropical Medicine, Nationalestraat 155, 2000 Antwerp, Belgium; Institut de Recherche Santé et Société, Université Catholique de Louvain, Clos Chapelle-aux-Champs, 30 boîte 3016-1200, Bruxelles, Belgique; Direction de la Recherche, Institut National de Santé Publique, avenue de l’Hôpital n°3, BP 6807 Bujumbura, Burundi; Belgian Health Care Knowledge Centre, Boulevard du Jardin Botanique 55, 1000 Brussels, Belgium; Ghent University, Coupure links 653, 9000 Ghent, Belgium; World Bank-Health Nutrition & Population (HNP), J Building-Room: 10-135 701 18th St NW, Washington, DC 20006 USA

**Keywords:** Performance-based financing, Malnutrition, Nutrition services, Impact evaluation, Research protocol, Mixed methods, Burundi

## Abstract

**Background:**

Malnutrition is a huge problem in Burundi. In order to improve the provision of services at hospital, health centre and community levels, the Ministry of Health is piloting the introduction of malnutrition prevention and care indicators within its performance based financing (PBF) scheme. Paying for units of services and for qualitative indicators is expected to enhance provision and quality of these nutrition services, as PBF has done, in Burundi and elsewhere, for several other services.

**Methods:**

This paper presents the protocol for the impact evaluation of the PBF scheme applied to malnutrition. The research design consists in a mixed methods model adopting a sequential explanatory design. The quantitative component is a cluster-randomized controlled evaluation design: among the 90 health centres selected for the study, half receive payment related to their results in malnutrition activities, while the other half get a budget allocation. Qualitative research will be carried out both during the intervention period and at the end of the quantitative evaluation. Data are collected from 1) baseline and follow-up surveys of 90 health centres and 6,480 households with children aged 6 to 23 months, 2) logbooks filled in weekly in health centres, and 3) in-depth interviews and focus group discussions. The evaluation aims to provide the best estimate of the impact of the project on malnutrition outcomes in the community as well as outputs at the health centre level (malnutrition care outputs) and to describe quantitatively and qualitatively the changes that took place (or did not take place) within health centres as a result of the program.

**Discussion:**

Although PBF schemes are blooming in low in-come countries, there is still a need for evidence, especially on the impact of revising the list of remunerated indicators. It is expected that this impact evaluation will be helpful for the national policy dialogue in Burundi, but it will also provide key evidence for countries with an existing PBF scheme and confronted with malnutrition problems on the appropriateness to extend the strategy to nutrition services.

**Trial registration:**

ClinicalTrials.gov PRS Identifier: NCT02721160; registered March 2016

## Background

### Malnutrition

Malnutrition is a huge problem worldwide. In 2010 about 115 million children worldwide were underweight, 55 million had low-weights-for-height and 171 million under the age of five years had stunted growth. Over the last decades, progress has been achieved. The proportion of children under the age of five years in developing countries who were underweight is estimated to have declined from 29 % to 18 % between 1990 and 2010. Yet, this rate was inadequate to meet the Millennium Development Goal 1, Target 1.C of halving levels of underweight between 1990 and 2015 [1].

Many of the challenges the global community faces in its fight against malnutrition are present in Burundi, one of the poorest countries in the World (with a GDP estimated at PPP $ 770 in 2014 [2]): 58 % of children under five years old suffer from chronic malnutrition and 6 % from acute malnutrition. Young children are particularly vulnerable: 10 to 11 % of the children aged 6–18 months were found to be acutely malnourished in 2010 [3].

These poor figures result from many factors. Food security is a major problem in the country. The majority of the population lives in rural areas. The high population density and overall poverty put pressure on land use, constrain investment and affect agricultural productivity. The landlocked nature of Burundi and its very weak economy also limit financial and geographic access to food markets. This results each year in food deficits: in 2009, the daily average food ratio was 1600 kcal per inhabitant (while FAO and WHO recommendations are between 2000 and 2600 kcal, depending on age and gender). Children from the poorest households stand greater risk to be undernourished, than their counterparts in the most privileged households.

Another constraint is the low performance of the health system in addressing malnutrition, both on prevention and care aspects. With its partners, the Ministry of Health (MoH) implemented in 2010 a national protocol [4] with a new care approach of acute malnutrition that proposes a plan of treatment and follow-up of malnourished children to be integrated within the formal health system (i.e. health centres (HC) and hospitals). However, to date, only one third of HCs and half of hospitals provide malnutrition care services. Another problem is the dependency on technical and financial partners, mainly UNICEF and the World Food Program (WFP) who provide nutritional inputs on an unstable basis^1^. There are also constraints at facility level, such as weak stock management capacities.

Before developing this research protocol, we conducted a rapid assessment of the services at the HC level. We observed an overall neglect of nutrition services at all levels and noticed a lack of knowledge among health workers (HW) involved in nutrition activities, limited motivation among them and among community health workers (CHW), as well as weak supervision (Nimpagaritse and Ntakarutimana, unpublished observations).

### Health care financing in Burundi: the key role of PBF

Malnutrition is of course only one of the many health challenges faced by the population of Burundi. Constraints are tight, as the government has very limited resources. Still, the government of Burundi made major efforts over the last years to raise the health of the poor and most vulnerable groups.

A key milestone was the removal of user fees for children under five and deliveries implemented in May 2006. In 2006, Burundi also piloted Performance-Based Financing (PBF) in three provinces. The strategy rapidly gained popularity and in April 2010, PBF was scaled up to the whole country. By doing so, Burundi became the second country in Africa to implement PBF in the health sector nationwide (after Rwanda) and the first to use the PBF system to correct the malfunctions related to the implementation of the national free health care policy [5].

Today, all public and most private non-profit health facilities in the country—HCs and hospitals—are covered by the national PBF scheme. The program is governed by the ‘*Cellule Technique FBP*’, a technical group of the MoH. The Burundian PBF system reflects the main health priorities of the country and rests on contractual arrangements involving different parties: every month health facilities report their activities related to the incentivised indicators; these reports are reviewed and validated at the province level and then sent to the *Cellule Technique FBP* which approves transfers of subsidies to the health facilities. The completion of this process takes between two and three months [6]. In addition, these activities are semi-annually randomly checked by community based organizations contracted to verify (via community surveys) the truthfulness of activities reported by HC [7]. Besides, qualitative indicators are used quarterly to assess the quality of the working environment and taken into account as bonuses or penalties for the amount of PBF subsidies transferred to each facility. The national PBF program is financed from several sources, with the Government and the World Bank (WB) being the largest and second largest sources of funding respectively.

There is a strong conviction in Burundi that the national PBF scale-up improved national health care financing, in terms of coherence, governance and vision [5]. Over the years, evidence has accumulated to support the policy [8–10], even if many questions remain. PBF has been put forward as a possible strategy to improve coverage rates of high impact interventions, quality of care, efficiency and overall performance of the health system [11–14]. PBF is a very recent policy innovation; the evidence base is growing, but is still very limited [15, 16]. A Cochrane review has concluded that the available evidence was too limited to ascertain the effectiveness of PBF in LMICs [17].

### PBF extended to nutrition indicators

#### Design and roll-out of the PBF nutrition intervention

To tackle the challenge of malnutrition and provide malnourished children with the most appropriate care, the MoH decided in 2013 to add nutrition indicators to the existing PBF scheme. A systemic strategy being needed, it was decided to incentivise three levels of services: the district hospitals, the HCs and the CHWs, on both prevention and care aspects, with a focus on children below five years old.

The PBF indicators related to nutrition follow the standard model in Burundi, and come as a combination of quantitative indicators to encourage an increase in service delivery and coverage rates (see Table 1) and qualitative indicators to stress the importance of improving the quality of services to ensure a positive outcome for the user [18]. Quality of nutrition activities is assessed quarterly, and a bonus or penalty is applied to subsidies received by the facilities according to their quality score: a facility with a score above 80 % will receive a bonus equivalent to the score multiplied by 25 % of the nutrition PBF subsidies; while a facility with a score below 60 % will be subject to a penalty of 10 to 25 % of the nutrition PBF subsidies. The verification of activities related to nutrition PBF indicators is also included in the usual verification system (see above).

**Table 1:** Quantitative indicators

Community level	(1) screening and referring of acute malnutrition cases to HCs, (2) organizing classes promoting good food and nutrition behaviours, and (3) organizing cooking practice classes.
Health centre level	(1) screening and caring severe and moderate acute malnutrition cases of children below five years old, or screening and referring acute malnutrition cases of children below five years old (depending on the type of HC^a^), and (2) growth follow-up and promotion for children below two years old.
Hospital level	(1) the number of treated severe acute malnutrition cases with medical complications of children below five years old and (2) the length of the stay at the hospital.

Note: ^a^Most of HCs do not provide care services for acute malnutrition cases and do have to refer cases to HCs that provide this type of care

Regarding the indicators at the community level, pre-verification of quantitative nutritional services provided is done by the HCs who supervise the CHWs, on the basis of registers and reports provided by them. The process of verification and validation is completed at the provincial level.

#### Implementation

It was jointly decided by the MoH and the WB that this addition of nutrition indicators to the existing PBF system deserved to be rigorously evaluated. The research design incorporates a randomized control component as far as HCs are concerned (see below for more details). It was decided that HCs in the control group would receive financial compensations equivalent to the PBF subsidies received in the intervention group (weighted average, considering volume of activities, staff).

**Fig. 1:**
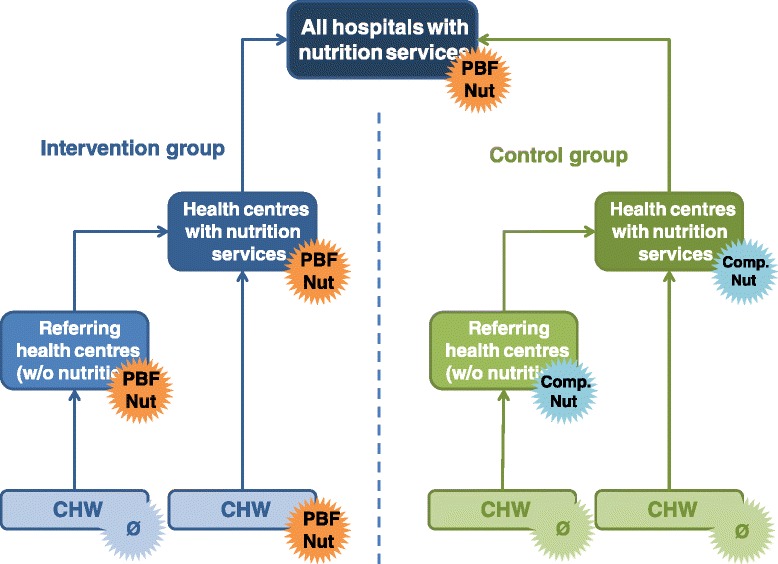
The PBF Nutrition Scheme. ‘PBF Nut’ means ‘Under PBF Nutrition programme besides the existing PBF scheme’ while ‘Comp Nut’ means input based financing compensation besides the existing PBF scheme

As shown in the Fig. 1, all hospitals with nutrition services fall under the Nutrition PBF program. Regarding HCs, we distinguish two types: nutritional HCs, i.e. HCs delivering the full package of nutrition services, and HCs that do not provide these services but instead refer acute malnutrition cases to the former. In the intervention group, both types of HCs are subject to the new nutrition indicators applied to PBF; while in the control group, both of them receive an input-based financing compensation. At the community level, the CHW groups that refer to the nutritional HCs are subject to the nutrition PBF in the intervention group while those in the control group will not receive any compensation due to budget constraints [18].

The intervention at nutritional HC and hospital levels started in January 2015, involving 45 nutritional HCs and 29 district hospitals with the extended PBF scheme, as well as 45 nutritional HCs with the compensation scheme (control group). The interventions in the other type of HCs as well as at the CHW level were expected to start in summer 2015, but due to major political instability since April 2015, they have started respectively only in January 2016 and in October 2015.

#### Theory of change

The introduction of nutrition activities into the PBF program translates policy makers’ belief that PBF can trigger some positive changes in the performance of the health personnel, facilities or system which will eventually impact on households and children. We have identified seven tracks for transmission of effects for the health facility performance:

1. **The income track**: the injection of extra financial resources might have a positive effect on nutrition services, as it allows the health facility manager to recruit more staff, to better equip his facility, etc.
2. **The cash track**: the fact that the financial resources are transferred directly to the health facility’s bank account allows the latter to rapidly and autonomously spend.
3. **The incentive track**: the extra resources are conditioned upon higher performance in nutrition activities; this should motivate community actors and staff to improve their performance in order to boost the health facility’s income and theirs (if bonuses are distributed among HWs). At facility level, the effect on other services is unclear: it can be negative for some (e.g. if the staff in charge of nutrition used to be responsible for other services which are now overlooked, as they are relatively less financially rewarding) and positive for others (if there are economies of scope—i.e. dedicating efforts to nutrition activities, reduce efforts required for other activities, thanks to synergies).
4. **The information track**: through the contract, the fee system and the related information sessions, staff have a clearer view on what performance should be, as far as nutrition services are concerned. Feedback from the program may also guide their decisions to improve. We hypothesize a positive effect on nutrition services. However, as for the incentive track, a negative effect could be that activities which are not remunerated may be perceived as non-important.
5. **Supervision & enforcement track**: under the new scheme, verification is extended to nutrition activities; this means that there will be more interaction between supervisors and the personnel in charge of nutrition. On top of the possible subsequent transfer of information (e.g. advice on good practices), the supervision may activate interpersonal motivators.
6. **Culture at provider level track**: a PBF scheme invites health facility managers to develop a work culture more favourable to innovation, flexibility, responsibility and entrepreneurship. As PBF has been a national policy for five years, one can assume that this is already the case in Burundi. However, one cannot exclude that it could positively influence the nutrition department more.
7. **Health system track**: it has been argued that PBF can trigger several system effects [16]. Here, part of these effects might come from the supervisors of the impact evaluation (e.g. the MoH requesting UNICEF and the WFP to better supply nutrition inputs; the WB solving some problems which may affect the study). Another part might come from the health facilities themselves (e.g. pressure upon the Department for Nutrition within the MoH to be a more reliable and responsive supplier). We expect that CHWs will refer more malnourished children to HCs and HCs will refer more SAM children to referral hospitals. This may trigger some unexpected feedback loops.

**Fig. 2:**
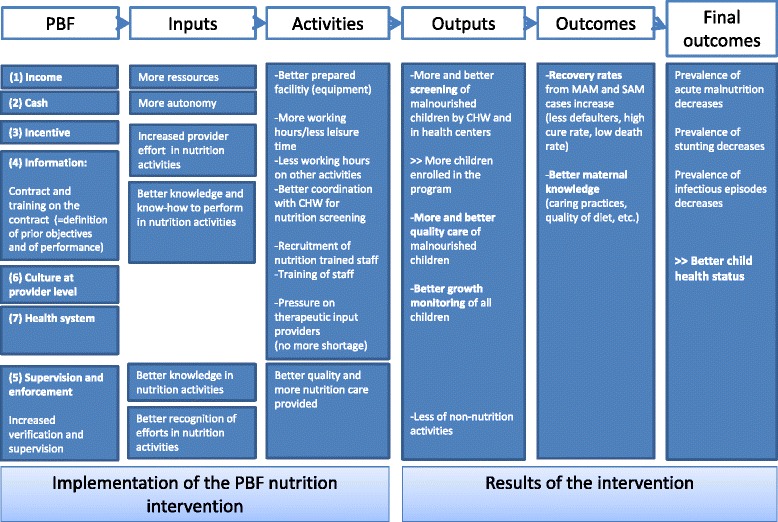
Results chain of the introduction of nutrition criteria in the PBF grid

For an overview of the expected results chain of the introduction of nutrition criteria in the PBF grid, see the Fig. 2.

There is also a specific theory of change for the control HCs. The main design difference between the intervention and control groups is the formula to obtain the extra funding: each HC of the intervention group has to improve its own performance to increase its revenue; control HCs do not have any effort to do: they just have to hope that the intervention group, as a whole, will be performing. This choice for the definition of the two groups to compare indicates that the main causal path under investigation is the incentive one. Other tracks are not denied (see the qualitative part of the research) but are considered as secondary Table 2.

**Table 2:** Routes applying in the Intervention and Control groups at the facility level

	Intervention group	Control group
(1) Income	++	++ ^a^
(2) Cash	++	++ ^a^
(3) Incentive	++ on nutrition services ? on other services	~
(4) Information	++	+ ^b^
(5) Supervision & enforcement	++	? ^c^
(6) Culture at provider level	~ or +	~ or + ^d^
(7) Health system	++	? ^e^

Notes: Symbols + and ++ mean that routes apply moderately to strongly; ? means unclear, ~ means neutral ^a^The control HCs will get additional financial resources which will correspond to a weighted average of the nutrition subsidies received in the intervention group. ^b^Before the start of the intervention, all 90 HCs were provided with some information on the nutrition indicators and on their performance in nutrition services. ^c^In a district where there are control and intervention HCs, the district team supervisors may transfer good practices to both intervention and control HCs. ^d^Control HCs know that there will probably be a scale up of the nutrition indicators to all HCs after the pilot; some managers may anticipate this by already reorganizing their nutrition services. ^e^Some health system effects might affect control HCs (e.g. if there is a problem of availability of nutritional inputs at national level, because of the pressure by intervention HCs, the supply to control HCs may be reduced)

## General objectives and research questions

The overarching objective of our research is to assess the effects of the introduction of criteria focusing on malnutrition prevention and care activities in the existing PBF system. Main research questions at 1) population and 2) HC levels are:

1. Does the introduction of criteria focusing on malnutrition prevention and care activities in the existing PBF system result in a reduction in acute and chronic malnutrition rates in the community?
2. Does the introduction of criteria focusing on malnutrition prevention and care activities in the existing PBF program result in better outcomes at the facility level, i.e. in better management of malnutrition cases (better recovery rates, shorter periods of treatment, etc.)?

In addition, the research incorporates two sub-questions which will allow identifying the possible causes of the success or any disappointing outcome, including unintended side-effects: Table 3

3) What are the transmission mechanisms induced by the introduction of criteria focusing on malnutrition prevention and care activities in the existing PBF program?
4) What are the reasons for the success or failure of PBF on nutrition activities?

**Table 3:** Main outcomes for the impact evaluation

Level	Outcomes
Population	- Prevalence of acute malnutrition (defined as WHZ < -2 or MUAC < 125 mm) and of stunting (defined as HAZ < -2) among children aged 6-23 months - WHZ, HAZ and MUAC among children aged 6-23 months
Health Centre	- Recovery rates from MAM and SAM cases of children below five years old - Duration of treatment of MAM and SAM cases of children below five years old

Notes: HAZ stands for Height-for-Age Z-score; MAM for Moderate Acute Malnutrition; MUAC for Mid-Upper Arm Circumference; SAM for Severe Acute Malnutrition; and WHZ for Weight-for-Height Z-score

In addition to these questions, the data collection has been designed in such a way to allow assessment of equity and efficiency of the intervention, potential externalities on other outcomes, and systemic spill-over effects, including possible demand for professional training by the health facilities. These aspects are not covered in this protocol paper.

## Method

This evaluative research adopts a mixed methods design which is increasingly seen as essential in health systems research as it allows researchers to view problems from multiple perspectives, contextualize information, and develop a more complete understanding of a problem [19]. It is also especially important in low- and middle-income country settings, where an in-depth understanding of social, economic and cultural contexts is essential to assess health systems’ performance [20]. With the increasing number of LMICs introducing PBF strategies, other researchers have used mixed methods to assess their impact [21–24].

### The quantitative component

Our main questions (1) and (2) require quantified measures and the possibility to attribute variations in the outcome measures to the intervention. We have opted for a *cluster-randomized controlled evaluation design*, with health centres^2^ as the primary unit of sampling and *sous-collines*^3^ (sub-hills) as the secondary unit of sampling. The 90 selected nutrition HCs were allocated to either a control group (45) or a treatment group (45). For each household survey and around each selected HC, six *sous-collines* are randomly selected; and in each *sous-colline*, 12 households with at least one child aged 6–23 months are surveyed.

#### Sample size and randomization

The sample size of the household surveys is computed on the smallest difference in the main outcome that can be considered of public health significance, i.e. a reduction of about 25 % in acute malnutrition prevalence in intervention centres as compared to control centres. Assuming that the intervention will result in decreasing the prevalence of MAM in children aged 6–23 months from 10 % to 7.5 % [3], and assuming that 65 children aged 6–23 months are to be surveyed in the catchment area of each HC, 90 HCs were needed to be randomized to either the intervention or control group, for an α-error of 5 % and a β-error of 20 %. The number of children per HC was increased to 72 to allow for missing or incomplete data, amounting to a total of 6,480 children aged 6–23 months over the 90 selected HCs. In total, a sample of 6,480 children were surveyed for the baseline, and, two years after the start of the program, another sample of 6,480 children aged 6–23 months will be surveyed.

Selection of HCs invited to participate in the study was done by simple randomization (computer-based random selection) among the 193 eligible HCs, i.e. HCs providing nutrition services (treatment of SAM and MAM). The 90 selected HCs have been paired on essential parameters of organization and functioning in relation to the outcomes (MAM rehabilitation activity, volume of activity, population in the catchment area, and percentage of recovery among malnourished children) as measured during the baseline survey. This will then be used to control for the potential confounding effect of these parameters. Within each of the 45 pairs, allocation to the intervention was done randomly through a lottery system organized by the MoH during a workshop in December 2014.

Simple randomization should be applied to select the households to be surveyed in the catchment area of the 90 HCs. However, such an approach was difficult logistically and could have turned out to be extremely resource-consuming. The chosen alternative option was to sample clusters of households, i.e. households living close to each other. Six clusters of 12 eligible households were selected at random from the whole catchment area of a given HC. This could be done without expanding the overall sample size given the quite high intra-class correlation considered for the sample computation.

The sample size of clinical files within the HC survey was computed on the smallest difference in the main outcome that could be considered of public health significance in intervention centres. Assuming that the intervention will result in increasing the recovery rate of acute malnutrition in children under five years from 80 % to 90 %, for an α-error of 5 %, a power of 80 % and an inter-cluster correlation (ICC) of 0.15 [25], a minimum of 12 clinical files per HC and per nutrition service (MAM and SAM rehabilitation services) were needed, among all children having been registered in the six previous months [26].

#### Data collection tools

The household survey focuses on question (1) and collects information on the nutritional and health status of each selected child, as well as general information on their household (including socio-economics, food security variables). The health facility survey focuses on questions (2), (3) and (4). To get information on malnutrition recovery rates, a total of 24 individual clinical files randomly selected among the files of all children below five years old enrolled in the MAM care program (12 files) and in the SAM care program (12 files) during the last six months are transcribed. In addition, organizational aspects of the HCs as well as of the nutrition services are recorded through interviews to managers. To assess the quality of services, we combine two techniques: patient-provider observations carried out on six paediatric consultations (performed by a maximum of two HWs) and exit interviews at the end of each of these observed consultations, in order to get information on the satisfaction level as well as to record anthropometrics of the children. Finally, to assess knowledge of the observed HWs, we use vignettes to measure the practical knowledge on different tasks to perform: a pattern of consultation is proposed and the health worker can ask all the questions (related to history and physical exams) necessary to arrive at a diagnosis and propose a treatment. Three vignettes are administered to every health worker observed in consultation.

#### Quality assurance plan and data management

During both survey rounds, lot quality assurance surveys are performed regularly by the field coordinator to assess accuracy of anthropometric data in the records. Most data are entered in “real-time”, and irregularities detected and corrected by the field coordinator on a continuous basis. Data entry is done with the use of an electronic device: Android smartphones with Open Data Kit software and the ONA internet data management platform have been chosen for this purpose^4^. The electronic data entry has the advantage of reducing risks of errors in recording the answers (thanks to automatic validity checks), and eliminating the need for double data entry from the paper to software transcription and to decrease considerably the time for transcription, estimated at 1,500 working days per survey round. Some questionnaires though need to be performed on paper (like the patient-provider consultation using an observation grid on paper): a double entry session is then organized to avoid any entry error.

#### Analysis strategy plan

First, some descriptive analysis are carried out in order to understand the main features of malnutrition management and of health services in general in Burundi at the HC level. Validation of the design was performed with the baseline survey data by comparing the treatment group with the control group.

Second, with both survey rounds’ data, the impact of the intervention will be assessed with multilevel statistical models with random effects at the HC level. Continuous dependent variables will be analysed in mixed-effect regression models, whereas categorical ones (e.g. recovery yes/no) will be analysed in logistic regression or Poisson regression models. At the population level, other factors of child malnutrition, such as household food security, socio-economic status, etc., will be controlled for. Interactions with season, child age and sex, stunting, and socio-economic parameters will be analysed. An equity analysis will also be performed in order to understand whether the intervention benefits more the poorer or richer households. At the HC level, other factors of child malnutrition recovery, such as for instance HC staff and CHWs knowledge and know-how, will be controlled for. Interactions with child age and sex, stunting and MUAC will be analysed.

### The qualitative component

Some of the answers to questions (3) and (4) will be informed by the facility survey. As it may not be enough, we will complement our quantitative results with some qualitative information. Qualitative data collection activities are intended to explain the heterogeneity across facilities (treatment and control) in relation to all outcomes observed quantitatively. This should lead to a more comprehensive understanding of the underlying causes compared to what would otherwise be possible through an exclusive use of quantitative methods. In other words, the qualitative component is shaped in a way to fill the knowledge gaps identified by the quantitative components [19].

Not only will we try to understand how the changes occur but also why. By documenting regularly, throughout the intervention, all the events related to the prevention and management of malnutrition, we will be able to know the organizational changes resulting from the PBF and in particular the various strategies undertaken at the HCs to improve their performance. We will also conduct in-depth interviews (IDI) and focus group discussions (FGD) to understand the quantitative performance data. We will be particularly attentive to understand the reasons why the HCs from the intervention group did not perform better than in the ‘control’ group if this happens to be the case; why some have not adopted effective strategies adopted by their peers; why some are clearly better than others; why HCs underperforming failed to catch up with the best performing HCs.

#### Sampling

Sampling approaches for the qualitative study component differ from those in the quantitative components. Both HCs and respondents will be sampled purposively.

First, to identify the various strategies that could be taken by HCs throughout the intervention, we propose a prospective follow-up with logbooks, in all 90 HCs, which allow a weekly report on the events related to the fight against malnutrition.

Second, IDIs and FGDs will be carried out among HCs selected purposively within the intervention group depending on the patterns observed in nutrition PBF routine data of each HC throughout the intervention. We will consider HCs representative of each pattern and qualitative data collection will be carried out until we reach saturation. Respondents will be selected using maximum variation sampling for each HC (the staff of HC, users, other stakeholders of the system).

Third, IDIs and FGDs will be carried out among HCs selected purposively within each group (intervention and control) depending on the changes observed through the quantitative impact evaluation in nutrition performance. Within each group, two sub-groups will be represented: the better and the less performing ones. We will consider as a starting point the best and the worst performing HCs and qualitative data collection will continue until we reach saturation. As above, respondents will be selected using maximum variation sampling for each HC.

#### Data collection tools

##### Logbooks

Our first strategy is prospective and rests on the participation of HC managers in the study group (both treatment and control groups). They have been entrusted a paper logbook which allows them to record some basic information on (1) bottlenecks (e.g. a stock-out of nutritional inputs), (2) extra financial or in-kind assistance received (from technical and financial partners such as NGOs) and (3) initiatives taken by the HC staff to improve performance of the nutrition services (e.g. meeting with the CHWs to improve the retrieval of children lost in follow-up, photocopies of management tools not provided by the central level). The health managers are requested to document the nutrition activities, inside the health facility, among the CHWs, and around the HC on a weekly basis.

The aim of this tool is to document changes at these three levels in order to track their influence on the performance of the health facility. Filled logbooks are collected on a monthly basis. It is expected to be helpful background documentation for the retrospective qualitative interviews.

##### In-depth interviews with key informants

IDIs will be conducted among profiles such as HC managers, HC staff, district-and province-level stakeholders. An interview guide will be developed and tested on the basis of the different components of the theory of change and information reported in the logbooks.

##### Focus group discussions

Around the same HCs, FGDs among CHWs may be performed. An interview guide will also be developed and tested on the basis of the different components of the theory of change and the information reported in the logbooks.

#### Quality assurance plan and data management

Regarding logbook data, close monitoring is performed so that they are effectively filled in weekly. The collection of fulfilled logbooks is done monthly via the provincial committee (along with their verification activities) in sealed envelopes.

IDIs with key informants will be conducted following precise rules ensuring the quality of data collected (permanent reflexivity, flexibility in the selection and construction of data collection tools, awareness of social relationships and feelings, triangulation) and transcription (making quick notes and transcript). We will be conducting ourselves in-depth interviews in French. FGDs will be conducted in the local language with the help of trained research assistants.

All verbal material (IDIs and FGDs) will be tape-recorded, fully transcribed, and translated into French when necessary for analysis. Transcripts and translations will be checked for content consistency and accuracy.

#### Analysis strategy plan

For the prospective study using the logbooks, data will be evaluated regularly from the end of the first year with a final analysis after the final impact analysis. For the retrospective ones, IDIs and FGDs will be performed first, after the analysis of PBF routine data of 2015, and second, after the preliminary impact analysis. Data will be transcribed and uploaded in the software Nvivo. Transcripts from interviews and focus group discussions will be coded applying thematic content analysis, which identifies recurrent themes that form a cluster of linked categories containing similar meanings. Analyst triangulation will be applied across all qualitative data sets. An additional valuable source of triangulation will be provided by comparing findings across data sources and across respondents (policy stakeholders, providers, and users). Figure 3 below displays the main stages of this research.

## Discussion

This protocol relates to the measurement and understanding of the impact of an extension of the PBF program in Burundi to nutrition indicators.

This is an ambitious research project, by the multifactorial nature of the health problem addressed, the complexity of the intervention, the number of facilities participating and the set of international and national partners involved in the intervention and the evaluation. Moreover, there is today limited knowledge on how a PBF system addressing malnutrition should function, and the impact of the intervention is not guaranteed. By the date of submitting this paper (May 2016), we can report that this evaluation has to deal with three other unexpected challenges.

The first one relates to the incomplete clinic patient files and registers that could affect the power of ex-post calculation of the impact assessment at the HC level. To address this, we must advocate for good filling in and archiving of these files and registers.

A second limitation concerns the very low qualification of personnel. The critical issue here would be to enable (if possible) the HWs to attend training personalized to their needs, or at least to the needs identified for the HC as a whole. HCs managers formally expressed their demand for training and their willingness to pay for them, according to a Vickrey process: we plan to organise training for the HCs who showed most interest.

A third and final concern is obviously the political situation since April 2015 which has inevitably consequences on the health system. Indeed, some donors have already announced the cancellation of their budget support. We should also pay attention to donor decisions on the supply of inputs for the MAM.

This will be the first assessment of the extension of an existing PBF scheme to another set of indicators. The mixed methods design rests on the awareness that understanding the processes through which health interventions produce change is as important as measuring the actual change produced. It follows that a comprehensive assessment of health interventions can therefore only be achieved by coupling quantitative methods with qualitative ones as part of a systematic mixed methods study design [27].

Moreover, at this stage, no African country has yet applied PBF to malnutrition (at least systematically), while malnutrition is a huge problem in many poor countries. A growing number of them are in the process of conducting pilot PBF projects. If the evaluation leads to conclusive results about the introduction of nutritional indicators in the PBF program, such a strategy could attract many countries of the continent (or even beyond). To the extent that these countries are already advanced in their PBF experience, the introduction of nutrition indicators can benefit from extremely fast dissemination.

We hope that this explanatory mixed methods design will be able to contribute to existing evidence by addressing the current knowledge gaps related to the effect of PBF models specifically with respect to the adding of new indicators to an existing PBF scheme.

## Abbreviations

CHW, Community Health Workers; FGDs, Focus Group Discussions; GDP, Gross Domestic Product; HAZ, Height-for-Age Z-score; HC, Health Centre; HIV/AIDS, Human immunodeficiency virus infection/acquired immune deficiency syndrome; ICC, inter-cluster correlation; IDI, in-depth interview; ITM, Institute of Tropical Medicine of Antwerp, Belgium; LMICs, low- and middle-income countries; MAM, Moderate Acute Malnutrition; MDGs, Millenium Development Goals; MoH, Ministry of Health; MSPLS, Ministère de la Santé Publique et de la Lutte contre le SIDA; MUAC, Mid-Upper Arm Circumference; PBF, Performance Based Financing; SAM, Severe Acute Malnutrition; UNICEF, Fonds des Nations unies pour l’enfance; UZA, University of Antwerp; WB, World Bank; WFP, World Food Programme; WHZ, Weight-for-Height Z-score

## Footnotes

1 For instance, because of budgetary constraints, in 2014, WFP stopped providing nutritional inputs in most of the country, making most moderate malnutrition care services ineffective.

2 This research at the health facility level focuses on HCs (and not hospitals) because the primary care level is a well-known bottleneck for the diagnosis and management of acute malnutrition.

3 *Sous-collines* are the smallest administrative entity in Burundi.

4 For more information on Open Data Kit software, cf. https://opendatakit.org/; and on ONA, cf. https://ona.io/home/
